# Event and Comment

**Published:** 1911-01-15

**Authors:** 


### DENTAL REGISTER.

Vol. LXV. January 15, 1911. No. 1

### EVENT AND COMMENT.

THE TAGGART PATENT AGREEMENT. In another place we publish in full a circular letter issued by the Board of Directors of the Dental Protective Association setting forth the terms of an agreement made with Dr. Wm. H. Taggart for the use of his process for making gold inlays and the purchase of his machine on a legal and definite basis. It seems strange that some such arrangement could not have been made before and saved all the controversy and bad feeling that has resulted from the introduction of this important technical procedure in dental practice. But some such experience seems to accompany the introduction of all important inventions, and our profession seems to have followed the rule and with scarcely an exception. It seems to us that the proposition is eminently fair to all parties and we trust the profession will unitedly embrace the opportunity to extend to Dr. Taggart such credit for his invention as he is entitled to, and compensate him adequately for the time and money which he has put into this invention and the production of a casting machine that leaves little to be desired technically considered. If the Dental Protective Association shall be the means of amicably adjusting this tangle its usefulness will again have been demonstrated, and Dr. Crouse will have put us under a new obligation for a generous and efficient service that few men would have undertaken or been able to achieve. Now let every dentist who is making use of this process make haste to accept one or the other of the propositions and not only settle this matter in a friendly and ethical manner, but at the same time revive the Dental Protective Association for some future good office which is certain to develop as time passes. We have made good use of this organization in the past and it may be capable of further usefulness. Perhaps it can induce Dr. Taggart to discontinue his suit.

ANNUAL MEETING OHIO STATE DENTAL SOCIETY. The forty-fifth annual meeting of this important Dental Society was held in the Great Southern Hotel at Columbus, Ohio, December sixth, seventh and eighth. As usual the meeting was largely attended and full of interesting events. The experiment of confining the programme to one phase of professional activity largely seems to have been a success. This is strong evidence that the profession is taking more interest in the preventive phase of its work than ever before, and that material progress in the rescue and preventive treatment in dentistry is surely being made. The clinics were numerous and the subjects treated were sufficiently varied to meet the needs of the most versatile and exacting In fact there were so many clinics that no one could see them all, and it seems that fewer clinics given in some way that all could have the advantage of seeing them should prove more instructive. The man who will work out a practicable method of giving clinical exhibits will do a grand service to the profession. The reorganization of the State into component societies has been very thoroughly and quickly done, and this State Society will be one of the most influential in the country. Much important business was done, including a donation of $1000 to the Miller Memorial Fund, $500 to the Oral Hygiene Committee, and an appropriation for the meeting of the National Dental Association at Cleveland next July. By a decisive vote the Society declined to change the date of its meeting to October or November. This should be done to early in January as the present time for holding this meeting is about as inconvenient as it could be; but it is hard to reform a bad habit.

ADOPTION OF THE METRIC SYSTEM IN DENTISTRY. The following resolution was adopted at the last meeting of the National Dental Association, and it seems to be the only way this important innovation can be promoted.

Resolved, That it is the conviction of the National Dental Association that the Metric System shall become the accepted standard of weights and measures in all professional and commercial transactions.

To accomplish this purpose at as early a date as may be practicable it is urged that all physicians, dentists, druggists and dealers in precious metals be urged to put into practice this system of weights and measures at once.

It is further resolved, That this resolution be published with the proceedings of the Society and a copy sent to all the Dental Journals and to the Secretary of the U. S. Pharmaceutical Convention, Dr. Murray Galt Motter, 1841 Summit Place, Washington, D. C.

Our present system of weights and measures for handling all dental commodities is not only complex, but is daily becoming more difficult and obsolete, because of the almost general adoption of the metric system in other parts of the world where dental activities are recorded in literature in the language of this system. It is so much simpler and easier to compute that scientific workers refuse to do their work, even in this country, in the old system. If our manufacturers and dealers in precious metals and other dental supplies would adopt it for all such transactions, we would soon learn it and like it so much that we would want to make all our calculations by the metric system. Let every one encourage his dealer to adopt it and use his influence with the large manufacturers to market their products on this basis.

